# The French glioblastoma biobank (FGB): a national clinicobiological database

**DOI:** 10.1186/s12967-019-1859-6

**Published:** 2019-04-23

**Authors:** Anne Clavreul, Gwénaëlle Soulard, Jean-Michel Lemée, Marion Rigot, Pascale Fabbro-Peray, Luc Bauchet, Dominique Figarella-Branger, Philippe Menei, FGB network, M. Boone, B. Chauffert, C. Desenclos, Y-E. Herpe, H. Sevestre, R. Tebbakha, O. Blanchet, A. Rousseau, S. Bouillot-Eimer, P. Dubus, H. Loiseau, K. Lacut, I. Quintin-Roué, R. Seizeur, L. Bekaert, D-H. Berro, F. Chapon, E. Emery, N. Rousseau, G. Ahle, R. Heller, M-C. Tortel, J. Voirin, M-H. Aubriot-Lorton, W. Farah, C. Schaeffer, E. Gay, S. Lantuejoul, P. Mossuz, B. Ghaleh, W. Lahiani, A. Marniche, Ph. Cornu, J-Y. Delattre, K. Mokhtari, M. Sanson, P. Niel, J. Pallud, P. Varlet, K. Mokhtari, D. Ricard, Y. Yordanova, A-L. Di Stephano, C. Horodyckid, C. Villa, P. Gele, C-A. Maurage, N. Reyns, F. Caire, F. Labrousse, V. Fermeaux, J. Feuillard, F. Ducray, N. Dufay, J. Guyotat, D. Meyronet, O. Chinot, H. Dufour, T. Graillon, P. Metellus, C. Gozé, V. Rigau, J-B. Frenel, O. Kerdraon, D. Loussouarn, J-F. Mosnier, F. Almairac, V. Bourg, F. Burel-Vandenbos, D-C. Chiforeanu, P-J. Le Reste, V. Quillien, B. Turlin, O. Langlois, F. Marguet, M. Quillard-Muraine, A-M. Bergemer-Fouquet, A. Decock-Giraudaud, I. Zemmoura

**Affiliations:** 1 Département de Neurochirurgie, CHU, 4 rue Larrey, 49 933 Angers Cedex 9, France; 2 CRCINA, INSERM, Université de Nantes, Université d’Angers, Angers, France; 3 Département Promotion, Direction de la Recherche, CHU Nantes, Nantes, France; 4 Département de Biostatistique, Epidémiologie, Santé Publique, CHU Nîmes, Nîmes, France; 5 Unité de recherche EA2415, Université de Montpellier, Montpellier, France; 6 Département de Neurochirurgie, Hôpital Gui de Chauliac, CHU Montpellier, Université de Montpellier, Montpellier, France; 7 Institut des Neurosciences de Montpellier INSERM U1051, Montpellier, France; 8 APHM, Hôpital de la Timone, Service d’Anatomie Pathologique et de Neuropathologie, Marseille, France; 9 Aix-Marseille Univ, CNRS, INP, Inst Neurophysiopathol, Marseille, France

**Keywords:** Glioblastoma, Biobank, Biological materials, Clinical data, Database

## Abstract

**Background:**

Glioblastomas (GB) are the most common and lethal primary brain tumors. Significant progress has been made toward identifying potential risk factors for GB and diagnostic and prognostic biomarkers. However, the current standard of care for newly diagnosed GB, the Stupp protocol, has remained unchanged for over a decade. Large-scale translational programs based on a large clinicobiological database are required to improve our understanding of GB biology, potentially facilitating the development of personalized and specifically targeted therapies. With this goal in mind, a well-annotated clinicobiological database housing data and samples from GB patients has been set up in France: the French GB biobank (FGB).

**Methods:**

The biobank contains data and samples from adult GB patients from 24 centers in France providing written informed consent. Clinical and biomaterial data are stored in anonymized certified electronic case report forms. Biological samples (including frozen and formalin-fixed paraffin-embedded tumor tissues, blood samples, and hair) are conserved in certified biological resource centers or tumor tissue banks at each participating center.

**Results:**

Clinical data and biological materials have been collected for 1087 GB patients. A complete set of samples (tumor, blood and hair) is available for 66%, and at least one frozen tumor sample is available for 88% of the GB patients.

**Conclusions:**

This large biobank is unique in Europe and can support the large-scale translational projects required to improve GB care. Additional biological materials, such as peritumoral brain zone and fecal samples, will be collected in the future, to respond to research needs.

## Background

Gliomas are the most common type of malignant primary brain tumors, and glioblastoma (GB) is the principal glioma diagnosed in adults. The reported annual age-adjusted incidence of GB ranges from 0.59 to 3.69 per 100,000 people, and the median age at diagnosis is 64 years [1]. Many advances have been made toward understanding GB biology, but the current standard of care for newly diagnosed GB, the Stupp protocol, has remained unchanged for over a decade [2, 3]. Patient survival remains poor with this protocol, with an overall survival (OS) of 68% at 1 year, a median OS of 12.8 months and a median progression-free survival of 7.4 months [4].

Attempts should be made to improve GB care, through large-scale translational research programs including modern-omics (including genomics, epigenomics, radiogenomics, transcriptomics, proteomics, and/or metabolomics) and artificial intelligence technologies, to improve our understanding of the etiology of GB and to identify new biomarkers for diagnosis, prognosis and treatment, facilitating the development of personalized and specifically targeted therapies. Such programs will require large numbers of biological samples, including, in particular, frozen tumor and blood samples associated with clinical data, if we wish to obtain statistically significant results.

In France, where the annual incidence of GB has been estimated at 3.3 per 100,000 people, Darlix et al. [5] indicated that frozen tumor samples were stored for only 32% of the 12,410 cases of GB recorded between 2006 and 2011. These samples were banked for healthcare purposes and are not therefore associated with the quality forms and informed consent forms (ICFs) required for use in research projects. Furthermore, they are not centralized and the complete clinical data for these samples are not recorded on anonymized and certified electronic case report forms (eCRFs). This highlights the importance of implementing standard operating procedures and developing active collaborations between professionals from all the disciplines involved, for the collection and storage, in a biobank, of biological materials and associated clinical data for GB patients. The French GB biobank (FGB) was developed for this purpose, following a call for tenders from the “Institut National du Cancer” (INCa) in 2012 [6]. This academic biobank is run along the lines of The Cancer Genome Atlas (TCGA) (http://www.cancergenome.nih.gov) created by the National Cancer Institute (NCI) of the National Institutes of Health (NIH) in 2005 [7, 8]. The FGB holds biological materials and data for adult patients with GB, and it is run with the support of neurosurgeons, neuropathologists, neuro-oncologists and biologists from 24 centers located throughout France. Its overall aim is to establish a repository of samples associated with clinical data from about 1500–2000 GB patients including epidemiological, imaging, tumor characteristic and follow-up data, together with a collection of classic biological samples (including frozen and formalin-fixed paraffin-embedded tumor tissues and blood samples), and original samples (including tumor tissue frozen in dimethyl sulfoxide (DMSO) for the development of cell- and animal-based GB models, macroscopically normal peritumoral brain zone tissues, hair and fecal samples) to support translational research projects and artificial intelligence technologies for GB. We describe here the operation of the FGB, its strengths and research opportunities with a view to increasing the international standing of this biobank.

## Methods

### Design of FGB

**Fig. 1:**
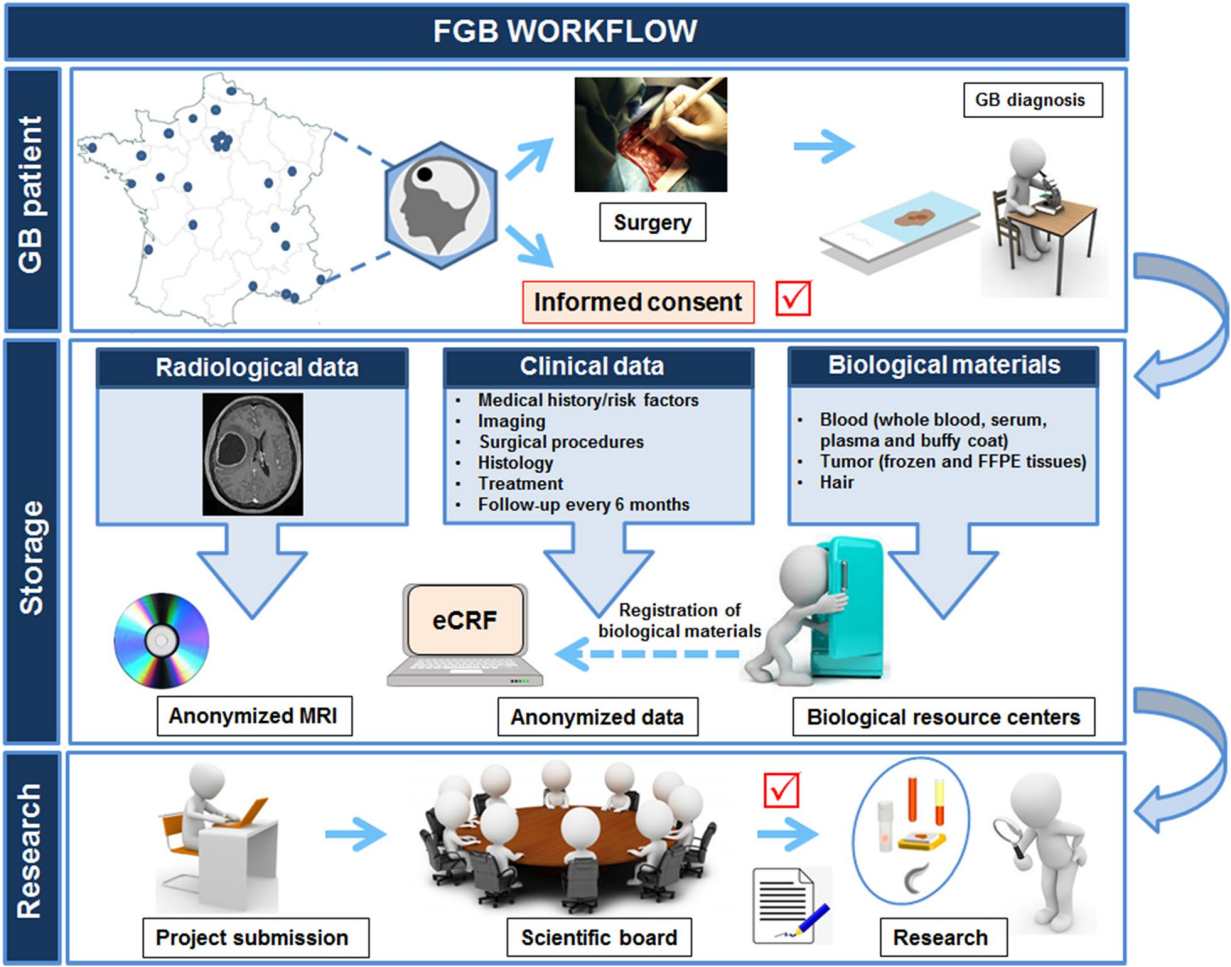
Presentation of FGB workflow. Only data and samples from adult patients with newly diagnosed GB who sign an informed consent form are included in the FGB. Clinical data and biological materials are registered in anonymized and certified eCRFs. Radiological data are accessible by electronic means or on an anonymized and coded CD-ROM. Biological materials, including blood, tumor and hair samples, are collected at the time of surgery and are stored in certified biological resource centers or tumor tissue banks at each participating center. Researchers wishing to use this collection complete a request form. If their request is accepted by the scientific board, a material transfer agreement is signed and the data and biological materials can be supplied

Following a call for tenders from INCa in 2012 (“Constitution de bases clinico-biologiques multicentriques nationales en cancérologie”), the FGB was set up, with the participation of 24 sites throughout France. The FGB is a member of BBMRI-ERIC (biobank number BB-0033-00093) and has a LinkedIn Page (https://www.linkedin.com/in/french-glioblastoma-biobank-808508153). The preparation of a website is currently underway. The legal and administrative aspects of this project are supported by Angers University Hospital. It is governed by a steering committee including representatives from all the participating centers and a scientific board including two or three members from each participating center. Figure 1 presents a schematic diagram of the workflow of data and biomaterial collection in the FGB.

### Eligibility and informed consent

The protocols and regulations of the FGB have been approved by the French Ministry of Health and Research (declaration number: DC-2011-1467, cession authorization number: AC-2017-2993), the CPP OUEST II ethics committee (CB 2012/02) and the CNIL (“Commission Nationale de l’Informatique et des Libertés”; the French national data protection authority; no. 1476342). All adult patients undergoing surgery for presumed primary GB are pre-included and asked to sign an ICF for the inclusion of their data and samples in the biobank. The ICF is also used for other brain tumor biobanks funded by INCa (http://www.e-cancer.fr). Patients are definitively included in the FGB when the diagnosis of GB is confirmed by the neuropathologist of the inclusion center according to the criteria of the 2016 CNS WHO classification [9]. A histology proofreading by a network of expert neuropathologists (RENOCLIP, “REseau National de Neuro-Oncologie CLInico-Pathologique”, France) may also be performed.

### Clinical data and biological material collection

The clinical data registered in the FGB include age, sex, family history of brain tumor, medical history, a short survey on pesticide, petrochemical and electromagnetic field exposure, date of symptoms and diagnosis, imaging data, Karnofsky performance status (KPS) score, tumor location, extent of resection, histology, standard biomarkers, treatments, and follow-up every 6 months until death.

Biological materials are collected at the time of surgery and include blood, tumor and a lock of hair (Table 1). The procedures for biological material collection and storage were developed in accordance with the best practice guidelines issued by several organizations, including INCa (http://www.e-cancer.fr), NCI (https://biospecimens.cancer.gov/bestpractices/2016-NCIBestPractices.pdf) and the International Society for Biological and Environmental Repositories (ISBER) [10]. They were adapted in line with the staff and organizational structures of the various participating center. Quality forms are used to ensure that the samples stored are fully traceable. Blood is collected into two serum-separation tubes (5 mL) and two EDTA tubes (6 mL), and transferred to the biobank for processing and storage. One of the EDTA tubes is used to prepare the whole-blood sample and the other tube is centrifuged for plasma recovery and buffy coat isolation. The two serum-separating tubes are also centrifuged, to prepare serum aliquots. All these samples are stored at − 80 °C. The lock of hair is stored in an envelope at room temperature. The resected GB tissue is immediately transported to the pathology department in an unfixed state. The pathologist takes the routine samples from the specimen required for diagnosis, and three samples (100–200 mg) of the tumor with > 40% tumor nuclei, excluding the periphery and necrosis, are collected and immediately frozen in liquid nitrogen. Freshly frozen GB samples are then stored at − 80 °C or in liquid nitrogen. A formalin-fixed, paraffin-embedded (FFPE) block of GB tissue is also prepared and stored at room temperature. The expression of the prognostic isocitrate dehydrogenase 1 (IDH1) R132H mutation is assessed, in all cases, by immunohistochemical and/or molecular genetic methods.

**Table 1:** Biological materials collected at the time of surgery and stored in the FGB

Biological materials	Quantity	Storage temperature
Blood		
Whole blood	500 µL (*n *= 1)	− 80 °C
Plasma	500 µL (*n *= 4)	− 80 °C
Serum	1 mL (*n *= 4)	− 80 °C
Buffy coats	250 µL (*n *= 2)	− 80 °C
GB tissue		
	100–200 mg/cryotube (*n *= 3)	− 80 °C or liquid nitrogen
	FFPE block (*n *= 1)	Room temperature
Hair		
	Lock (*n *= 1)	Room temperature

Other biomarkers are evaluated (the choice of biomarkers depending on the center): glial fibrillary acidic protein (GFAP), oligodendrocyte transcription factor 2 (Olig2), neurofilament, epidermal growth factor receptor (EGFR), p53, the Ki67/MIB1 proliferation marker, alpha internexin (INA) and alpha-thalassemia/mental retardation syndrome X-linked (ATRX), the loss of heterozygosity on chromosomes 1p, 19q, 10q, and 16, EGFR amplification, O^6^-methylguanine-DNA methyltransferase (MGMT) methylation, gains of chromosome 7 and Verhaak signatures.

### Biological material and data storage

All biological materials are stored in certified biological resource centers (NF96900 and/or ISO9001) or tumor tissue banks at the corresponding participating center. Clinical and biological material data are stored in anonymized and certified eCRFs built with Ennov Clinical software (Ennov, Paris, France). This software is ISO9001:2015-certified for all products and activities and meets the recommendations of the FDA 21CRF Part11 and EMA for the IT security of clinical data. Secure Sockets Layer is used to secure data transfer. Only authorized persons from each center can complete the eCRF (https://nantes-lrsy.hugo-online.fr/CSOnline). Quality control for data recording is based on pre-testing and consistency checks during data entry and data management. Initial imaging data are stored as a digital imaging and communications in medicine (dicom) format file in the picture archiving and communication system (PACS) of each center or on an anonymized and coded CD-ROM.

### Research proposals and the use of samples

The scientific board of the FGB ensures that the clinical data and/or biological materials collected are used for high-quality research projects. Researchers have to complete a request form that is submitted to the scientific board for acceptance or rejection within 4 weeks. If the request is accepted, material transfer agreements are signed between research institutions under the laws and regulations in force, and the data and/or samples are made available.

## Results

### Characteristics of the GB patients included in the FGB

**Fig. 2:**
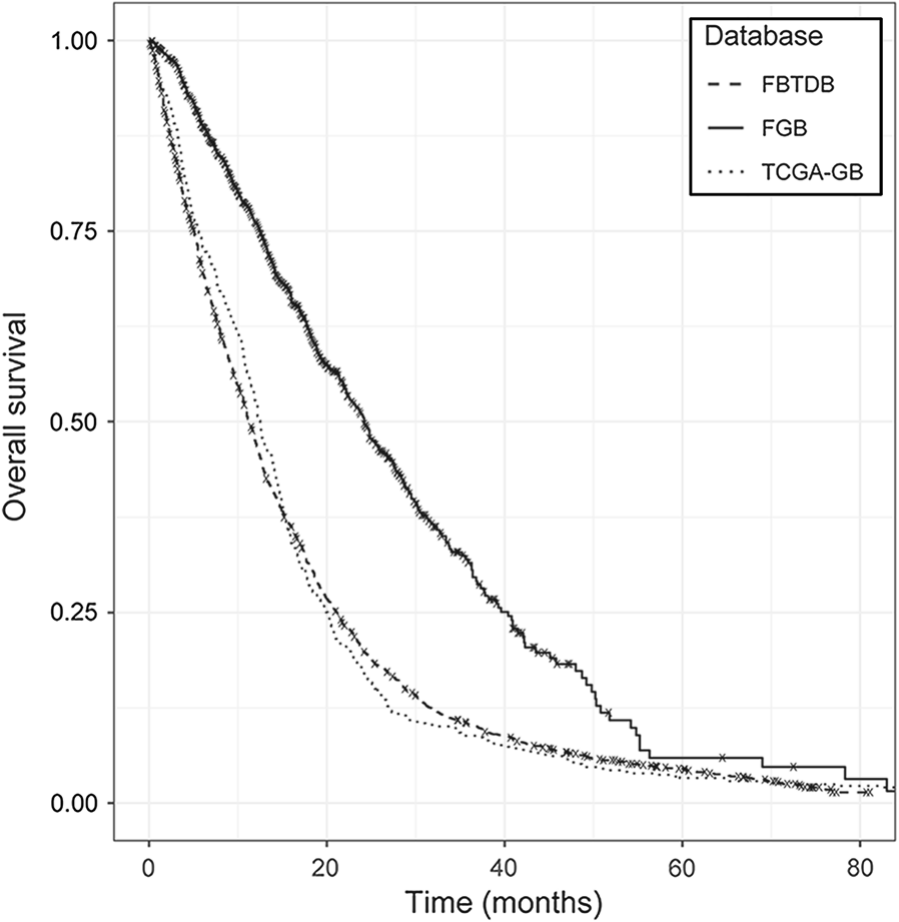
Comparison of the OS of the GB patients included in the FGB (*n *= 915) with that in two other GB cohorts: TCGA-GB (*n *= 486) and the French brain tumor database (FBTDB) (*n *= 1936). Survival curves were plotted according to the Kaplan–Meier method. Log-rank tests were performed to compare patient OS between the different cohorts with R v3.5.1 (https://www.r-project.org). Median OS is significantly longer for FGB patients than for the patients from TCGA-GB or FBTDB cohort (P < 0.001)

To date, data and samples for 1087 patients have been included in the FGB. For 573 patients, histology proofreading was performed by RENOCLIP and the initial diagnosis of GB was validated in 99.5% of cases. The baseline characteristics of the 1087 patients included are shown in Table 2. The median age at diagnosis is 63 years for these patients, and the incidence of the disease is higher in men than in women (sex ratio = 1.5). The GB included are unilobar or multilobar, located mostly in the supratentorial region (frontal, temporal, parietal, and occipital lobes), and much more rarely in the cerebellum. The IDH1R132H mutation was detected in about 6% of FGB patients. MGMT promoter methylation status was analyzed in 38% of the GB patients and methylation of the MGMT promoter was detected in 49% of these patients. Data for familial and/or epidemiologic risk factors were collected for 62% of the GB patients, and about 18% indicated exposure to risk factors, such as pesticides (pesticide exposure reported for 44% of these patients). Median OS for the patients included in the FGB (*n *= 915) is 24.2 months. This median OS is significantly longer than that for the patients in two other GB cohorts: TCGA-GB (http://www.cancergenome.nih.gov/; *n *= 486; 12.5 months) and the French brain tumor database (FBTDB; *n *= 1936; 11.2 months) [11] (*P* < 0.001) (Fig. 2).

**Table 2:** Characteristics of the 1087 GB patients included

Patients included in the FGB	Number	%
Patient characteristics		
Age (median age: 63 years)		
< 70 years	801	73.7
≥ 70 years	286	26.3
Sex		
Male	653	60.1
Female	434	39.9
KPS score		
≥ 70	601	55.3
< 70	73	6.7
Missing data	413	38.0
Risk factors		
No relevant exposure	549	50.5
Relevant exposure	124	11.4
Family history of brain tumors	46	4.2
Electromagnetic fields	19	1.7
Petrochemical exposure	16	1.5
Pesticide exposure	55	5.1
Other (e.g. chemical products, asbestos, lead)	42	3.9
Missing data	414	38.1
OS		
Median (24.2 months)	915	84.2
Short (< 6 months)	100	9.2
Long (> 3 years)	61	5.6
Missing data	172	15.8
Tumor characteristics		
Tumor location		
Unilobar	454	41.8
Frontal lobe	166	15.3
Temporal lobe	169	15.5
Parietal lobe	91	8.4
Occipital lobe	18	1.7
Corpus callosum	8	0.7
Cerebellum	2	0.2
Multilobar	485	44.6
Missing data	148	13.6
2016 WHO CNS classification		
GB IDH-wildtype	937	86.2
Giant cell GB	35	3.2
Gliosarcoma	8	0.7
GB IDH-mutant	62	5.7
GB NOS	88	8.1
MGMT status		
Unmethylated	209	19.2
Methylated	201	18.5
Missing data	677	62.3
First-line treatment		
Type of surgery		
Biopsy	145	13.3
Surgical biopsy	35	3.2
Stereotactic biopsy	106	9.8
Missing data	4	0.4
Resection [total, subtotal (≥ 90%) or partial (< 90%)]	797	73.3
Lobectomy	9	0.8
Missing data	136	12.5
Surgical technique/per-operatory treatment		
Awake craniotomy	67	6.2
5-ALA fluorescence	78	7.2
Gliadel wafer	67	6.2
Adjuvant therapy		
Without adjuvant therapy	46	4.2
Radiotherapy alone	25	2.3
Radiotherapy/TMZ	624	57.4
Avastin (± radiotherapy/TMZ)	45	4.1
Gliadel wafer (± radiotherapy/TMZ)	65	6.0
Other chemotherapy	18	1.7
Missing data	177	16.3
Second-line treatment		
Surgery (± radio/chemotherapy)	105	9.7
Treatment without surgery	545	50.1

*5-ALA* 5-aminolevulinic acid,* IDH* isocitrate dehydrogenase,* KPS* Karnofsky performance status, *MGMT* O^6^-methylguanine-DNA methyltransferase,* NOS* not otherwise specified,* OS* overall survival

### Characteristics of the biological materials stored in the FGB

#### Type and number of samples stored in the FGB

**Fig. 3:**
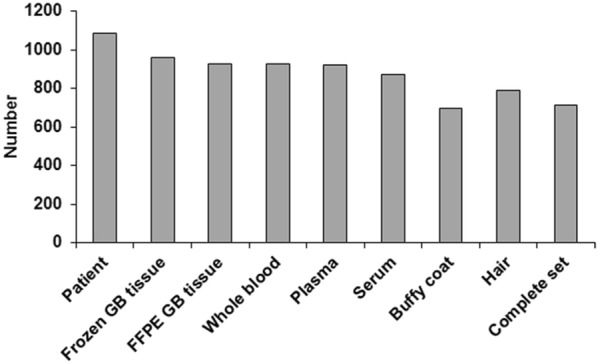
Number of samples collected at the time of primary surgery and stored in the biobank, by sample type. Complete set = tumor + blood + hair

Figure 3 indicates the number of samples stored, by sample type, for the 1087 patients included in the FGB. For 714 GB patients (66%), a complete set of samples (tumor, blood and hair) is available, and at least one frozen tumor sample is available for 961 GB patients (88%).

#### Procurement and storage of tumor tissue and blood samples in the FGB

Quality forms have been established to track blood and tumor tissue samples at each step, from sampling to storage. Tumor and blood samples are sent from the operating room to biological resource centers or tumor tissue banks at room temperature or at 4 °C in a pneumatic tube or vehicle, depending on the operating procedures of the center concerned. The interval between sample collection and freezing varies between centers, with a mean value of 2.4 ± 1.4 h (range: 30 min–26 h) for tumor tissue and 3.9 ± 0.4 h (range: 10 min–26 h) for blood samples. If the handling time for tumor tissues exceeds 3 h, a commercial RNA-stabilizing buffer (RNAlater) is added to preserve tissue quality during transport.

### The activities of the FGB

**Fig. 4:**
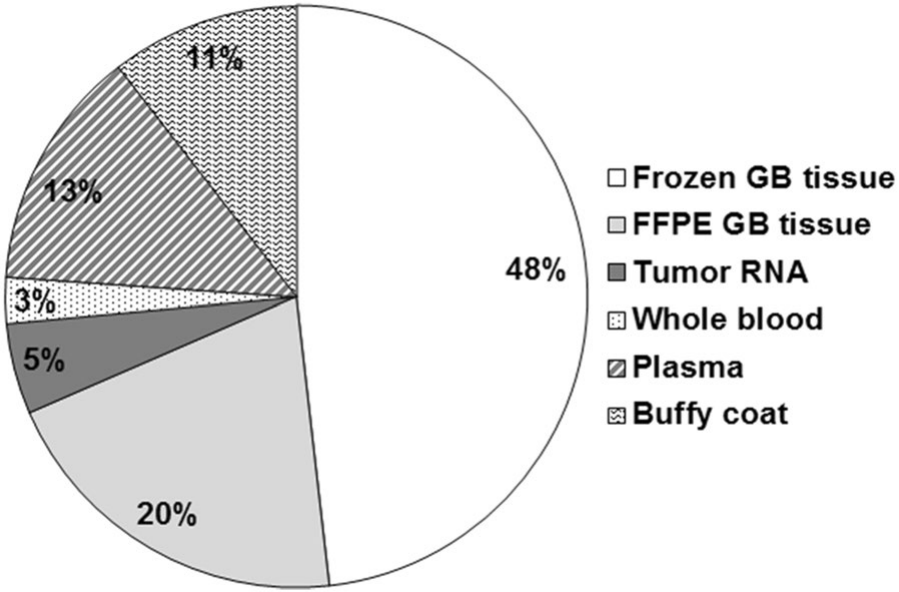
Types of samples requested

Nineteen request forms from public institutes or private companies have been submitted to the scientific board of the FGB. Twelve projects were accepted, one was refused and six are underway. Different types of samples have been requested, with frozen and FFPE tumor samples the most frequently sought (Fig. 4). The results from a project characterizing extracellular vesicles from the plasma of GB patients were recently published [12].

## Discussion

GB biobanks are an essential tool for ensuring an adequate supply of the high-quality samples associated with clinical data required to support translational programs and artificial intelligence technologies to foster the introduction of personalized medicine for this brain disease. Several glioma biobanks including GB tissues have been developed, in China and the USA, for example, but, to our knowledge, the FGB is the only national clinicobiological database for GB in Europe [7, 13–17].

### Strengths of the FGB

To date, the FGB has included data for 1087 adult GB patients, for 66% of whom a complete set (tumor, blood and hair) was obtained. The GB patients of the FGB are representative of the general population undergoing surgery for GB. In particular, GB is primarily diagnosed in older individuals, at a median age of 63 years, and the incidence of GB is higher in men than in women [1]. Consistent with other studies, the IDH1R132H mutation is rare in the GB patients of the FGB cohort, in which it was found in 6% of GB cases [18, 19]. Median OS is 24.2 months in the GB patients of the FGB, which is significantly longer than the median OS of the patients from two other GB cohorts (TCGA-GB and FBTDB). This difference can be explained by the more recent establishment of the FGB, as GB management has evolved since the other two cohorts were set up. In particular, advanced surgical techniques have been developed for optimal safe resection. Another possible explanation is that the FGB cohort contains only a small percentage of patients who have undergone biopsy (13.3% vs. 41.2% in the FBTDB) [11] because this surgical procedure results in the collection of insufficient tumor tissue for cryopreservation.

The FGB collects original samples, such as locks of hair, in addition to classic tumor and blood samples. This biomaterial is proposed as a non-conventional matrix for the biological monitoring of pesticide exposure [20]. In a preliminary study, we analyzed the levels of 60 pesticides from the organochlorine, organophosphorus, and pyrethroid families in the scalp fat of three GB patients. Three of the compounds tested, all from the organochlorine family, which have been banned in most countries, including France, for several years, were detected at high concentrations in the hair of all three patients: hexachlorobenzene (HCB), hexachlorocyclohexane beta (HCHB) and 4,4′ dichlorodiphenyldichloroethylene (4,4′DDE). The possible link between brain tumors and pesticides has been investigated over the last few decades, but remains controversial [21, 22]. The collection of hair samples included in the FGB provides a potential source of information about the influence of pesticides on the process of GB development.

In addition to the collection of clinical and biological data, imaging data can be obtained electronically or on an anonymized and coded CD-ROM. The accessibility of the FGB imaging data and biological materials will facilitate radiogenomic studies seeking to assess correlations between imaging findings and molecular markers predictive of treatment response and prognosis by noninvasive methods. We plan to upload radiological images onto the anonymized eCRF website, to improve data transmission.

The patient characteristics included in the FGB highlight some rare cohorts of interest, such as patients with professional pesticide exposure (*n *= 55), patients with a family history of brain tumors (*n *= 46), patients under the age of 40 years (*n *= 56), patients undergoing awake craniotomy (*n *= 67), patients with Gliadel wafers implanted during first-line surgery (*n *= 67), patients with long (> 3 years; *n *= 61) or short (< 6 months; *n *= 100) survival and patients with tumor samples frozen both at the time of primary surgery and during a relapse (*n *= 26). These samples are rare because surgery is the standard of care for newly diagnosed GB, but its role in the treatment of recurrent GB remains a matter of debate [23]. Furthermore, tumor samples are not systematically frozen during surgery for relapse. The analysis of these tumor samples will provide a comprehensive picture of recurrent GB of potential value for determining the best molecular targets.

### Quality assurance for the GB tissue and blood samples in the FGB

Certification of the FGB by an external international body has not yet been requested, but several procedures have been set up in the FGB to guarantee the quality of biological materials. Firstly, histological proofreading is performed via RENOCLIP to confirm an absence of overlap in microscopic appearance between the GB and other neoplastic lesions, such as pleomorphic xanthoastrocytoma and ganglioglioma [24]. Secondly, procedures for biological material collection and storage with associated quality forms have been implemented in the FGB. Particular attention was paid to the conditions for handling and storing fresh GB tissues. As formalin fixation results in RNA fragmentation, GB samples should be frozen in addition to being stored as FFPE blocks. Frozen samples can be used with different molecular tools, such as RNA sequencing, facilitating a comprehensive analysis of gene expression patterns. It is generally recommended to snap-freeze tumor samples as soon as possible after resection, and ideally within 30–60 min of resection. However, it is recognized that this may not be practically possible for a number of reasons, including the availability of pathology services, theater procedures and logistic issues [25, 26]. In the FGB, the time interval from excision to snap freezing is generally less than 2 h. The mean time lag to freezing can be explained, at most centers, by the absence of a neuropathologist in the operating room to examine and freeze tumor fragments directly, and a lack of pneumatic tube systems for the rapid transport of GB samples to the pathology laboratory. The time interval between excision and freezing, and the conditions in which tissues are maintained during this interval are well-annotated on the quality forms, so researchers receiving samples from this biobank are fully informed about the quality status of the samples they receive. The quality control data collected from end-users preparing derivatives for their studies have, to date, indicated high quality, with minimal RNA degradation in the samples received. Thirdly, FGB samples are stored in certified biological resource centers (NF96900 and/or ISO9001) or tumor tissue banks at each of the participating centers, to ensure the high-quality storage of biological materials. Samples derived from blood are stored at − 80 °C and frozen GB tissues are stored at − 80 °C or in liquid nitrogen, depending on the equipment available at the centers. We have not performed quality control tests on DNA, RNA, and protein at different time points for a subset of cases to check the integrity of the biological materials over time. Several studies have shown that long-term storage at − 80 °C is satisfactory and does not negatively influence RNA quality [26–28]. It remains unclear whether storage in liquid nitrogen has significant advantages over storage at − 80 °C. These data indicate that the storage conditions used for the FGB may be suitable for the long-term preservation of GB tissues and blood samples.

### Materials recently added to the FGB

**Fig. 5:**
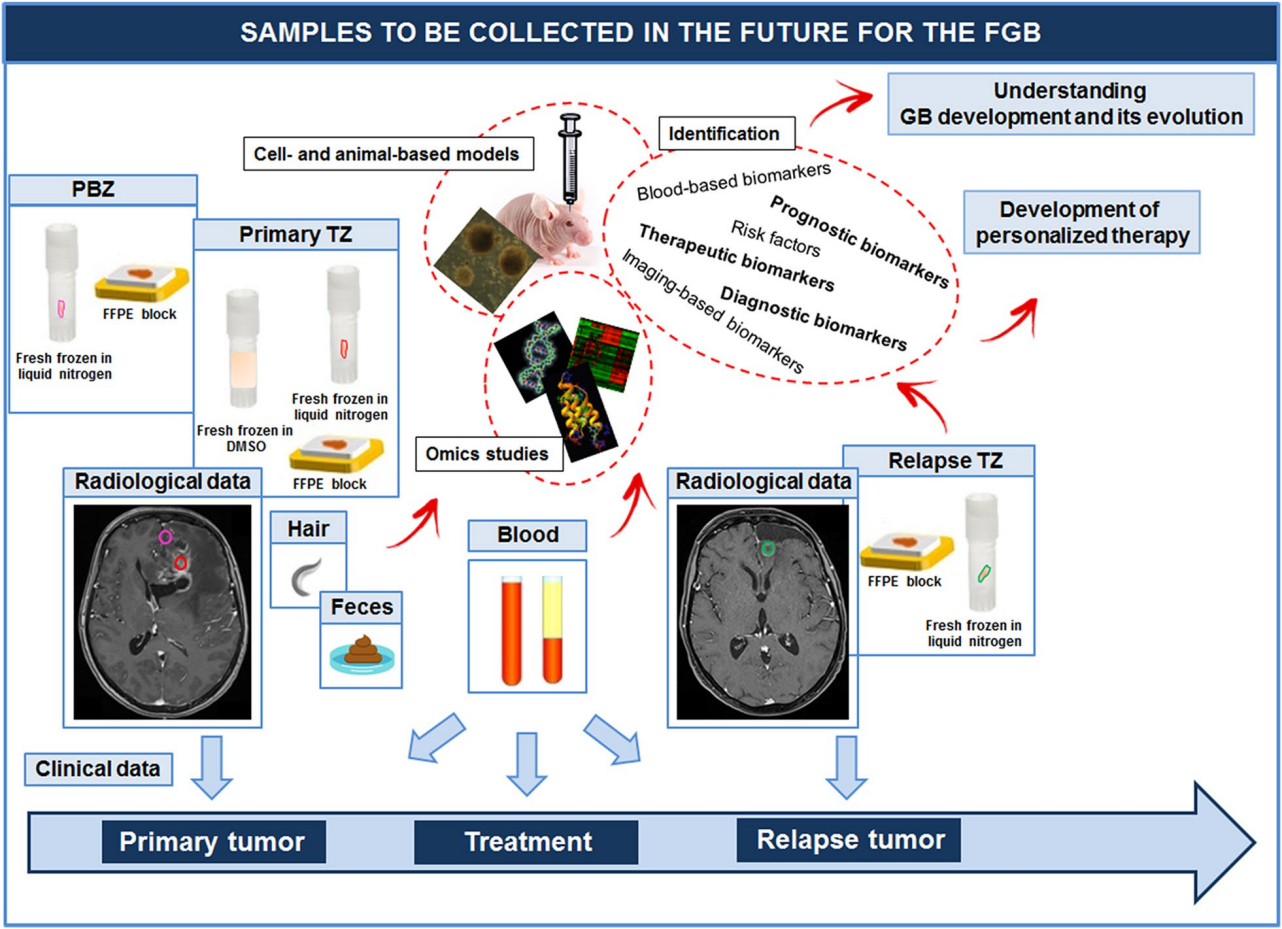
Materials recently added to the FGB. Since 2019, PBZ samples and tumor tissue frozen in DMSO at the time of primary surgery have been added to the FGB. Blood samples are also collected before surgery and during patient follow-up. Fecal samples will be collected in the near future. All these biological materials will support translational programs for understanding the development of GB and its progression and/or for identifying new diagnostic, prognostic and therapeutic biomarkers for the development of personalized therapy

Tumor samples are currently stored in the FGB in the frozen state and as FFPE blocks. Since 2019, fresh GB tissues have been frozen in DMSO for the development of cell- and animal-based GB models (Fig. 5). Glioma stem-like cells (GSLCs), also called glioma-initiating cells, generated from GB tissue are considered a more representative and reliable cell model than standard cell lines [29, 30]. Furthermore, patient-derived orthotopic xenograft models, obtained by engrafting GSLCs into immunocompromised mice, resemble human GB more faithfully [30, 31]. The demand for these preclinical GB models is growing exponentially, and tumor tissues frozen in DMSO and GSLCs will be required to satisfy this demand. Xie et al. [32] recently created a biobank of GSLCs called the human GB cell culture resource. We now include fresh tumor tissues dissociated in cell suspension and then frozen in DMSO in the FGB, to enable research scientists to develop cell- and animal-based models of GB.

In addition to the samples of GB tissues collected, macroscopically normal peritumoral brain zone (PBZ) tissues are now collected during primary surgery and stored in the FGB (Fig. 5). Many studies have analyzed the core of GB tumors, but only a few have focused on the PBZ, even though 90% of recurrences occur in this area [33]. We previously demonstrated the complexity of this zone, which has a degree of interpatient variability similar to that of the corresponding tumor zone [34–37]. The PBZ displays specific alterations, such as the presence of selected tumor clones and stromal cells with tumorigenic and angiogenic properties [38–43]. Characterization of the cellular and molecular components of the PBZ is required, to identify new molecular targets for the development of personalized targeted therapy and adjuvant treatments for GB after surgery. Advances in our knowledge of PBZ characteristics will also facilitate the development of approaches to refine the per-operative evaluation of this zone, to optimize surgical resection of the tumor. A large collection of PBZ samples is essential for such studies, and the FGB will serve this purpose.

Blood samples are also now collected before and during patient follow-up, for the identification of blood biomarkers for use in prognostic assessments, therapeutic monitoring, and surveillance for recurrence (Fig. 5). Three main classes of blood biomarkers have been evaluated in GB—extracellular macromolecules, extracellular vesicles, and circulating tumor and immune cells—but none was considered to constitute a clinically meaningful indicator of GB [44–46]. Much work remains to be done to identify blood biomarkers suitable for use in clinical practice. Samples collected before surgery and during follow-up are therefore essential and are now stored in the FGB. It has been suggested that cerebrospinal fluid (CSF) analysis would be a more appropriate way to screen for circulating biomarkers, as marker concentrations may be higher in the CSF than in the blood, given that the CSF circulates within the brain, on the other side of the blood–brain barrier. However, CSF collection is an invasive process involving lumbar puncture to obtain serial CSF samples from patients with GB. Blood collection and approaches aiming to identify biomarkers in the serum are therefore prioritized in the FGB.

In addition to locks of hair, we plan to collect, in the near future, fecal samples, another original type of sample not routinely collected in this context. In a recent proteome analysis [47], we observed an enrichment in “pathogenic *Escherichia coli* infection” in patients with GB. The collection of fecal samples will make it possible to define the role of gut microbiota composition in GB development and response to treatment, as has been done in other cancers, such as colorectal cancer, hepatocellular carcinoma and breast cancer [48].

### Sustaining the FGB

As shown by the experience of other biobanks, the development and maintenance of clinicobiological databases is a costly activity. The budget from the “Direction Générale de l’Offre des Soins” (DGOS) of the French Ministry of Health allocated to certified biological resource centers or tumor tissue banks covers the costs of collecting and storing biological materials, but not those of collecting and storing clinical data or managing these processes. The budget from different contracts signed with academic and industrial partners requesting samples for research projects is also insufficient to cover these costs. Additional funding must therefore be found to sustain such structures, and this process is time-consuming and fastidious, with low success rates. In the future, research institutions and governments must find solutions to facilitate the funding of clinicobiological databases. Furthermore, it will be essential to gain and maintain the trust and commitment of all participating centers to sustain such structures. A scientific board has been set up for the FGB, to select, according to transparent rules, the scientific projects authorized to use the data and biomaterial collected. A newsletter is published every 6 months, to inform all FGB staff about the number of inclusions and the projects using the stored data. Furthermore, a group called “FGB network”, including at least three representatives from each center has been set up and will be cited in all publications (in authors or acknowledgements) in which FGB samples are used, to enable centers to benefit from their own cooperative effort.

## Conclusions

Researchers and scientists wishing to set up large-scale studies for understanding the development of GB and its progression, and/or for identifying new diagnostic, prognostic and therapeutic biomarkers for the development of personalized therapy should contact the FGB (https://www.linkedin.com/in/french-glioblastoma-biobank-808508153) to ask for biological materials and associated clinical data. This academic biobank has already included samples and data for 1087 GB patients, and is flexible enough to adapt to diverse requests, making it possible to implement projects quickly.

## Abbreviations

CSF: cerebrospinal fluid
DMSO: dimethyl sulfoxide
eCRF: electronic case report form
EGFR: epidermal growth factor receptor
FBTDB: French brain tumor database
FFPE: formalin-fixed, paraffin-embedded
FGB: French glioblastoma biobank
GB: glioblastoma
GSLCs: glioma stem-like cells
ICF: informed consent form
IDH1: isocitrate dehydrogenase 1
INCa: “Institut National du Cancer”
KPS: Karnofsky performance status
MGMT: O^6^-methylguanine-DNA methyltransferase
NCI: National Cancer Institute
NIH: National Institutes of Health
OS: overall survival
PBZ: peritumoral brain zone
RENOCLIP: “REseau National de Neuro-Oncologie CLInico-Pathologique”
RNAlater: RNA-stabilizing buffer
TCGA: The Cancer Genome Atlas
TZ: Tumoral zone

## References

[1] Ostrom QT, Bauchet L, Davis FG, Deltour I, Fisher JL, Langer CE (2014). The epidemiology of glioma in adults: a “state of the science” review. Neuro-Oncology.

[2] Stupp R, Mason WP, van den Bent MJ, Weller M, Fisher B, Taphoorn MJB (2005). Radiotherapy plus concomitant and adjuvant temozolomide for glioblastoma. N Engl J Med.

[3] Stupp R, Hegi ME, Mason WP, van den Bent MJ, Taphoorn MJB, Janzer RC (2009). Effects of radiotherapy with concomitant and adjuvant temozolomide versus radiotherapy alone on survival in glioblastoma in a randomised phase III study: 5-year analysis of the EORTC-NCIC trial. Lancet Oncol.

[4] Kelly C, Majewska P, Ioannidis S, Raza MH, Williams M (2017). Estimating progression-free survival in patients with glioblastoma using routinely collected data. J Neurooncol.

[5] Darlix A, Zouaoui S, Rigau V, Bessaoud F, Figarella-Branger D, Mathieu-Daudé H (2017). Epidemiology for primary brain tumors: a nationwide population-based study. J Neurooncol.

[6] Menei P, Figarella-Branger D, Bauchet L, Loiseau H, Denyset M, Roman T (2014). French research infrastructures to develop and validate glioma biomarkers. Neurosurgery.

[7] Clark K, Vendt B, Smith K, Freymann J, Kirby J, Koppel P (2013). The Cancer Imaging Archive (TCIA): maintaining and operating a public information repository. J Digit Imaging.

[8] Tomczak K, Czerwińska P, Wiznerowicz M (2015). The Cancer Genome Atlas (TCGA): an immeasurable source of knowledge. Contemp Oncol Poznan Pol.

[9] Louis DN, Perry A, Reifenberger G, von Deimling A, Figarella-Branger D, Cavenee WK (2016). The 2016 World Health Organization Classification of Tumors of the Central Nervous System: a summary. Acta Neuropathol (Berl)..

[10] 2012 best practices for repositories collection, storage, retrieval, and distribution of biological materials for research international society for biological and environmental repositories. Biopreserv Biobank. 2012;10:79–161. 10.1089/bio.2012.1022 24844904

[11] Fabbro-Peray P, Zouaoui S, Darlix A, Fabbro M, Pallud J, Rigau V (2019). Association of patterns of care, prognostic factors, and use of radiotherapy-temozolomide therapy with survival in patients with newly diagnosed glioblastoma: a French national population-based study. J Neurooncol.

[12] André-Grégoire G, Bidère N, Gavard J (2018). Temozolomide affects extracellular vesicles released by glioblastoma cells. Biochimie.

[13] Aibaidula A, Lu J, Wu J, Zou H, Chen H, Wang Y (2015). Establishment and maintenance of a standardized glioma tissue bank: Huashan experience. Cell Tissue Bank.

[14] Bregy A, Papadimitriou K, Faber DA, Shah AH, Gomez CR, Komotar RJ (2015). Banking brain tumor specimens using a University core facility. Biopreserv Biobank.

[15] Cancer Genome Atlas Research Network (2008). Comprehensive genomic characterization defines human glioblastoma genes and core pathways. Nature.

[16] Fouke SJ, Benzinger TL, Milchenko M, LaMontagne P, Shimony JS, Chicoine MR (2014). The comprehensive neuro-oncology data repository (CONDR): a research infrastructure to develop and validate imaging biomarkers. Neurosurgery.

[17] Kong F, Zhang W, Qiao L, Li Q, Li H, Cao J (2018). Establishment and quality evaluation of a glioma biobank in Beijing Tiantan Hospital. PeerJ.

[18] Parsons DW, Jones S, Zhang X, Lin JCH, Leary RJ, Angenendt P (2008). An integrated genomic analysis of human glioblastoma multiforme. Science.

[19] Yan H, Parsons DW, Jin G, McLendon R, Rasheed BA, Yuan W (2009). IDH1 and IDH2 mutations in gliomas. N Engl J Med.

[20] Béranger R, Hardy EM, Dexet C, Guldner L, Zaros C, Nougadère A (2018). Multiple pesticide analysis in hair samples of pregnant French women: results from the ELFE national birth cohort. Environ Int.

[21] Fallahi P, Foddis R, Cristaudo A, Antonelli A (2017). High risk of brain tumors in farmers: a mini-review of the literature, and report of the results of a case control study. Clin Ter.

[22] Camille C, Ghislaine B, Yolande E, Clément P, Lucile M, Camille P (2017). Residential proximity to agricultural land and risk of brain tumor in the general population. Environ Res.

[23] Wann A, Tully PA, Barnes EH, Lwin Z, Jeffree R, Drummond KJ (2018). Outcomes after second surgery for recurrent glioblastoma: a retrospective case-control study. J Neurooncol.

[24] Gokden M (2017). If it is not a glioblastoma, then what is it? A differential diagnostic review. Adv Anat Pathol.

[25] Morente MM, Mager R, Alonso S, Pezzella F, Spatz A, Knox K (1990). TuBaFrost 2: standardising tissue collection and quality control procedures for a European virtual frozen tissue bank network. Eur J Cancer Oxf Engl.

[26] Song SY, Jun J, Park M, Park SK, Choi W, Park K (2018). Biobanking of fresh-frozen cancer tissue: RNA is stable independent of tissue type with less than 1 hour of cold ischemia. Biopreserv Biobank.

[27] Caixeiro NJ, Lai K, Lee CS (2016). Quality assessment and preservation of RNA from biobank tissue specimens: a systematic review. J Clin Pathol.

[28] Shabihkhani M, Lucey GM, Wei B, Mareninov S, Lou JJ, Vinters HV (2014). The procurement, storage, and quality assurance of frozen blood and tissue biospecimens in pathology, biorepository, and biobank settings. Clin Biochem.

[29] Lenting K, Verhaak R, Ter Laan M, Wesseling P, Leenders W (2017). Glioma: experimental models and reality. Acta Neuropathol (Berl).

[30] Patrizii M, Bartucci M, Pine SR, Sabaawy HE (2018). Utility of glioblastoma patient-derived orthotopic xenografts in drug discovery and personalized therapy. Front Oncol.

[31] Joo KM, Kim J, Jin J, Kim M, Seol HJ, Muradov J (2013). Patient-specific orthotopic glioblastoma xenograft models recapitulate the histopathology and biology of human glioblastomas in situ. Cell Rep.

[32] Xie Y, Bergström T, Jiang Y, Johansson P, Marinescu VD, Lindberg N (2015). The human glioblastoma cell culture resource: validated cell models representing all molecular subtypes. EBioMedicine.

[33] Lemée J-M, Clavreul A, Menei P (2015). Intratumoral heterogeneity in glioblastoma: don’t forget the peritumoral brain zone. Neuro-Oncol.

[34] Aubry M, de Tayrac M, Etcheverry A, Clavreul A, Saikali S, Menei P (2015). From the core to beyond the margin: a genomic picture of glioblastoma intratumor heterogeneity. Oncotarget.

[35] Com E, Clavreul A, Lagarrigue M, Michalak S, Menei P, Pineau C (2012). Quantitative proteomic Isotope-Coded Protein Label (ICPL) analysis reveals alteration of several functional processes in the glioblastoma. J Proteomics.

[36] Lemée J-M, Com E, Clavreul A, Avril T, Quillien V, de Tayrac M (2013). Proteomic analysis of glioblastomas: what is the best brain control sample?. J Proteomics.

[37] Lemée J-M, Clavreul A, Aubry M, Com E, de Tayrac M, Eliat P-A (2015). Characterizing the peritumoral brain zone in glioblastoma: a multidisciplinary analysis. J Neurooncol.

[38] Clavreul A, Etcheverry A, Chassevent A, Quillien V, Avril T, Jourdan M-L (2012). Isolation of a new cell population in the glioblastoma microenvironment. J Neurooncol.

[39] Clavreul A, Guette C, Faguer R, Tétaud C, Boissard A, Lemaire L (2014). Glioblastoma-associated stromal cells (GASCs) from histologically normal surgical margins have a myofibroblast phenotype and angiogenic properties. J Pathol.

[40] Clavreul A, Etcheverry A, Tétaud C, Rousseau A, Avril T, Henry C (2015). Identification of two glioblastoma-associated stromal cell subtypes with different carcinogenic properties in histologically normal surgical margins. J Neurooncol.

[41] Glas M, Rath BH, Simon M, Reinartz R, Schramme A, Trageser D (2010). Residual tumor cells are unique cellular targets in glioblastoma. Ann Neurol.

[42] Piccirillo SGM, Dietz S, Madhu B, Griffiths J, Price SJ, Collins VP (2012). Fluorescence-guided surgical sampling of glioblastoma identifies phenotypically distinct tumour-initiating cell populations in the tumour mass and margin. Br J Cancer.

[43] Rampazzo E, Della Puppa A, Frasson C, Battilana G, Bianco S, Scienza R (2014). Phenotypic and functional characterization of glioblastoma cancer stem cells identified through 5-aminolevulinic acid-assisted surgery [corrected]. J Neurooncol.

[44] Figueroa JM, Carter BS (2018). Detection of glioblastoma in biofluids. J Neurosurg.

[45] Holdhoff M, Yovino SG, Boadu O, Grossman SA (2013). Blood-based biomarkers for malignant gliomas. J Neurooncol.

[46] Zachariah MA, Oliveira-Costa JP, Carter BS, Stott SL, Nahed BV (2018). Blood-based biomarkers for the diagnosis and monitoring of gliomas. Neuro-Oncol.

[47] Lemée J-M, Clavreul A, Aubry M, Com E, de Tayrac M, Mosser J (2018). Integration of transcriptome and proteome profiles in glioblastoma: looking for the missing link. BMC Mol Biol.

[48] Rea D, Coppola G, Palma G, Barbieri A, Luciano A, Del Prete P (2018). Microbiota effects on cancer: from risks to therapies. Oncotarget.

