# Correction to: The French glioblastoma biobank (FGB): a national clinicobiological database

**DOI:** 10.1186/s12967-020-02345-5

**Published:** 2020-04-30

**Authors:** Anne Clavreul, Gwénaëlle Soulard, Jean-Michel Lemée, Marion Rigot, Pascale Fabbro-Peray, Luc Bauchet, Dominique Figarella-Branger, Philippe Menei, FGB network, M. Boone, B. Chauffert, C. Desenclos, Y-E. Herpe, H. Sevestre, R. Tebbakha, O. Blanchet, A. Rousseau, S. Bouillot-Eimer, P. Dubus, H. Loiseau, K. Lacut, I. Quintin-Roué, R. Seizeur, L. Bekaert, D-H. Berro, F. Chapon, E. Emery, N. Rousseau, G. Ahle, R. Heller, M-C. Tortel, J. Voirin, M-H. Aubriot-Lorton, W. Farah, C. Schaeffer, E. Gay, S. Lantuejoul, P. Mossuz, B. Ghaleh, W. Lahiani, A. Marniche, Ph. Cornu, J-Y. Delattre, K. Mokhtari, M. Sanson, P. Niel, J. Pallud, P. Varlet, K. Mokhtari, D. Ricard, Y. Yordanova, AL. Di Stefano, C. Horodyckid, C. Villa, P. Gele, C-A. Maurage, N. Reyns, F. Caire, F. Labrousse, V. Fermeaux, J. Feuillard, F. Ducray, N. Dufay, J. Guyotat, D. Meyronet, O. Chinot, H. Dufour, T. Graillon, P. Metellus, C. Gozé, V. Rigau, J-B. Frenel, O. Kerdraon, D. Loussouarn, J-F. Mosnier, F. Almairac, V. Bourg, F. Burel-Vandenbos, D-C. Chiforeanu, P-J. Le Reste, V. Quillien, B. Turlin, O. Langlois, F. Marguet, M. Quillard-Muraine, A-M. Bergemer-Fouquet, A. Decock-Giraudaud, I. Zemmoura

**Affiliations:** 1 Département de Neurochirurgie, CHU, 4 rue Larrey, 49 933 Angers Cedex 9, France; 2 CRCINA, INSERM, Université de Nantes, Université d’Angers, Angers, France; 3 Département Promotion, Direction de la Recherche, CHU Nantes, Nantes, France; 4 Département de Biostatistique, Epidémiologie, Santé Publique, CHU Nîmes, Nîmes, France; 5 Unité de recherche EA2415, Université de Montpellier, Montpellier, France; 6 Département de Neurochirurgie, Hôpital Gui de Chauliac, CHU Montpellier, Université de Montpellier, Montpellier, France; 7 Institut des Neurosciences de Montpellier INSERM U1051, Montpellier, France; 8 APHM, Hôpital de la Timone, Service d’Anatomie Pathologique et de Neuropathologie, Marseille, France; 9 Aix-Marseille Univ, CNRS, INP, Inst Neurophysiopathol, Marseille, France

## Correction to: J Transl Med (2019) 17:133 10.1186/s12967-019-1859-6

Following publication of the original article [1], the authors identified an error in the name of one of the collaborators from the FGB network, AL. Di Stefano.

The incorrect name of the collaborator is: A-L. Di Stephano.

The correct name of the collaborator is: AL. Di Stefano.

The list of collaborators has been updated in the list of collaborators below and in the metadata of this correction.
